# Adenosquamous Carcinoma of the Pancreas: Outcomes of a Multicenter European Study (ADESQUPAN Project)

**DOI:** 10.1245/s10434-025-19051-5

**Published:** 2026-01-27

**Authors:** Jose Manuel Ramia, Celia Villodre, Dimitrios Giakoustidis, Paraskevi Chatzikomnitsa, Pietro Addeo, Philippe Bachellier, Gennaro Nappo, Alessandro Zerbi, Julie Navez, Gerardo Blanco-Fernández, Jakob Kirkegård, Juli Busquets, Bergthor Björnsson, Santiago Sánchez-Cabús, Elizabeth Pando, Sebastian Manuel Staubli, Sanjay Pandanaboyana, Daniel Aparicio-López, Harry V. M. Spiers, Paola Melgar, Rami Rhaiem, Maria João Amaral, Cristina Vallejo-Bernad, Bodil Andersson, Fernando Burdío, George Tzimas, Isabel Mora-Oliver, Michael Rousek, Carlos Domingo-del-Pozo, Ahmad Mahamid, Dimitrios Lytras, Evangelos D. Lolis, Rafael López-Andújar, María Sorribas, Pablo Alejandro López, Hassan Elghonemy, Bhargava Chikkala, Weiboom Lim, Anita Balakrishnan, María Villamonte Román, Cristina Ballester, Carlos Hörndler-Algárate, Mario Serradilla-Martín

**Affiliations:** 1 Department of Surgery, Hospital General Universitario Dr. Balmis, Alicante, Spain; 2 ISABIAL: Instituto de Investigación Sanitaria y Biomédica, Alicante, Spain; 3 Universidad Miguel Hernández, Alicante, Spain; 4 Department of Surgery, Papageorgiou General Hospital, Thessaloniki, Greece; 5 Department of Surgery, University of Strasbourg, Strasbourg, France; 6 Pancreatic Surgery Unit, Department of Biomedical Sciences, Humanitas Clinical and Research Centre, IRCCS, Humanitas University, Milan, Italy; 7 Department of Abdominal Surgery and Transplantation, Hôpital Universitaire de Bruxelles (HUB), Université Libre de Bruxelles (ULB), Brussels, Belgium; 8 Department of Surgery, Instituto Universitario de Investigación Biosanitaria de Extremadura, School of Medicine, Hospital Universitario de Badajoz, University of Extremadura, Badajoz, Spain; 9 Department of Surgery, HPB Section and Institute for Clinical Medicine, Aarhus University Hospital, Aarhus University, Aarhus, Denmark; 10 Department of Surgery, Hospital Universitario de Bellvitge, Hospitalet de Llobregat, Barcelona, Spain; 11 Department of Surgery in Linköping and Department of Biomedical and Clinical Sciences, Linköping University, Linköping, Sweden; 12 Department of Surgery, Hospital Universitario de la Santa Creu i Sant Pau, Barcelona, Spain; 13 Department of Surgery, Hospital Universitario Valla d’Hebron, Barcelona, Spain; 14 Department of Surgery, Royal Free Hospital, London, UK; 15 Department of Surgery, Freeman Hospital, Newcastle Upon Tyne, UK; 16 Department of Surgery, Hospital Universitario San Jorge, Huesca, Spain; 17 Department of Surgery, Cambridge University Hospitals, Cambridge, UK; 18 Department of Surgery, CHU Reims, Reims, France; 19 Department of Surgery, Unidade Local de Saúde de Coimbra, Coimbra, Portugal; 20 Department of Surgery, Hospital Universitario San Pedro, Logroño, Spain; 21 Department of Clinical Sciences Lund, Surgery, Lund University and Skane University Hospital, Lund, Sweden; 22 Department of Surgery, Hospital de Mar, Barcelona, Spain; 23 Department of Surgery, Hygeia Hospital, Athens, Greece; 24 Liver, Biliary and Pancreatic Surgery Unit, Department of General and Digestive Surgery, Department of Surgery, Biomedical Research Institute, INCLIVA, Hospital Clínico Universitario de Valencia, University of Valencia, Valencia, Spain; 25 Department of Surgery, Second Faculty of Medicine of Charles University, Military University Hospital, Prague, Czech Republic; 26 Department of Surgery, Hospital Universitario Dr. Peset, Valencia, Spain; 27 HPB Surgery Unit, Department of Surgery, Rappaport Faculty of Medicine, Carmel Medical Center, Technion Israel Institute of Technology, Haifa, Israel; 28 Department of Surgery, General Hospital of Volos, Thessalia, Greece; 29 Department of Surgery, General Hospital of Chania, Crete, Greece; 30 Department of Surgery, Hospital Universitario y Politécnico La Fe, Valencia, Spain; 31 Department of Pathology, Hospital Universitario Miguel Servet, Zaragoza, Spain; 32 Department of Surgery, Instituto de Investigación Biosanitaria Ibs.GRANADA, School of Medicine, Hospital Universitario Virgen de Las Nieves, University of Granada, Granada, Spain

**Keywords:** Ad enosquamous, Pancreas, Cancer, Surgery

## Abstract

**Background:**

Adenosquamous carcinoma of the pancreas (ASCP) is a rare and aggressive subtype of pancreatic cancer. Compared with other pancreatic tumors, ASCP has a notably poorer prognosis and shorter survival rates. The optimal therapeutic approaches to ASCP have yet to be defined.

**Methods:**

This retrospective, multicenter European study included all consecutive patients who underwent elective pancreatic surgery for ASCP at participating centers between 2010 and 2024. The inclusion criteria encompassed all patients who underwent scheduled surgery for ASCP during the study period. The exclusion criteria ruled out patients without a confirmed pathologic diagnosis of ASCP, those who did not undergo surgery, and patients with extra-pancreatic disease.

**Results:**

The study analyzed 194 patients from 29 hospitals in 11 European countries. The overall survival rates were 56.2% at 1 year, 26.3% at 3 years, and 9.8% at 5 years. The disease-free survival rates at the same intervals were 36.6%, 16.5%, and 6.7%, respectively. In the multivariate analysis, significant associations with shorter survival were R2 resections, lymphatic stromal invasion, T4 stage, no adjuvant chemotherapy, and recurrence.

**Conclusions:**

Patients who undergo resection for ASCP experience very low 5-year survival rates (10%). It is advisable to avoid resecting T4 tumors in patients with significant comorbidities or R2 resections. Additionally, failure to provide adjuvant chemotherapy, often due to severe postoperative complications, further deteriorates the prognosis.

Adenosquamous carcinoma of the pancreas (ASCP) is a rare aggressive variant combining glandular and squamous differentiation. Although ASCP traditionally is defined by at least 30% squamous components, this threshold is debated due to its subjectivity.^1–10^ First described in 1907 and known by several names, ASCP is distinguished from metastatic squamous cell carcinoma of the pancreas by the presence of glandular elements.^6,11^

Several hypotheses explain ASCP histogenesis,^1,2,4,10,11^ including the differentiation theory currently favored due to shared genomic alterations that suggests both components originate from the same progenitor cells. The squamous metaplasia theory proposes that the squamous component develops from pre-existing pancreatic ductal adenocarcinoma (PDAC), whereas the collision theory is largely dismissed^4^. Immunohistochemically, ASCP typically shows positivity for CK5/6, CK7, and p63, and negativity for CK20, p16, and p53.^4^ Loss of chromosome 9p21 also is linked to poorer prognosis.^12^

Representing 0.5–4% of pancreatic cancers,^1,2,5,7–10^ ASCP often occurs in the pancreatic tail.^1,10,11,13,14^ It typically is larger and poorly differentiated and has more lymph node involvement than PDAC.^1,10,11,13,14^ Diagnosed at about the age of 68 years, mostly in males, some cases show hypercalcemia.^1,3^ Preoperative diagnosis without tissue is rare,^1,7^ but computed tomography (CT)-based radiomics may help differentiate ASCP from PDAC.^15^

As an understudied disease, ASCP has no clearly defined therapeutic strategies^1,5^. Surgical resection is recommended when feasible.^1,2,6,10,11,16^ The prognosis for ASCP is poorer than for PDAC,^1–3,5,7,9,11^ with a 2-year survival of about 30%, a 5-year survival of 7%, and a median survival of 7–11 months.^2,3,5,8,13^

Our study aimed to assess the incidence, prevalence, and current management of ASCP in a European series. This will provide a better understanding, offer clinical recommendations, and identify prognostic and risk factors that predict worse overall survival (OS) and disease-free survival (DFS).

## Methods

### Patients and Study Design

This retrospective, multicenter European study included all consecutive patients who underwent elective pancreatic surgery for ASCP at participating centers between 1 January 2010 and 30 June 2024. The study followed Strengthening the Reporting of Observational Studies (STROBE) guidelines.^18^

### Inclusion and Exclusion Criteria

The inclusion criteria encompassed all patients who underwent scheduled surgery for ASCP during the study period. The exclusion criteria ruled out patients without a confirmed pathologic diagnosis of ASCP, those who did not undergo surgery, and patients with extra-pancreatic disease.

### Definitions

Patients’ comorbidities were summarized using the Charlson Comorbidity Index^19^ and the American Society of Anesthesiologists (ASA) score classification.^20^ Postoperative complications were assessed and classified using the Clavien-Dindo classification of surgical complications as well as the Comprehensive Complication Index (CCI).^21,22^ Major complications were defined as Clavien-Dindo grade IIIa or higher. Resection margins, including transection and circumferential margins, were classified as R0 (distance from margin to tumor ≥1 mm), R1 (distance from margin to tumor < 1 mm), or R2 (macroscopically positive margin) according to the Royal College of Pathologists’ definition.^23^ The original histopathologists’ report was accepted. Tumors were staged using the tumor-node-metastasis (TNM) classification.^24^

Complications, readmissions, and mortality were recorded up to 90 days postoperatively. Specific complications such as post-pancreatectomy hemorrhage, biliary leakage, delayed gastric emptying, and pancreatic fistula were categorized according to the definitions of the International Study Group of Pancreatic Surgery (ISGPS).^25–28^

### Data Collection

Each participating center appointed a dedicated contact person responsible for all communication with the study coordinator. This person received login codes and passwords for the online electronic case report form (eCRF) environment, specifically REDCap (Research Electronic Data Capture, Vanderbilt University, Nashville, TN, USA). Each data collector was provided a separate login account, and the chief study coordinators could monitor all activity.

### Ethics

Approval was obtained from the Aragon Research Ethics Committee (CEICA, C.I. PI24/012, 24/01/2024). All data were collected anonymously, without patient identifiers. Participating centers were instructed to link the patient's local medical record numbers to an anonymous study patient ID. Informed consent was waived because there was no associated risk. All actions were logged following Good Clinical Practice (GCP) guidelines.

### Statistical Analysis

Data were analyzed using R software (R-2.14.1; The R Foundation for Statistical Computing, Vienna, Austria, 2011). The statistical tests used included Student’s *t* test, Mann–Whitney *U* test, chi-square test, and Fisher’s exact test as appropriate. Categorical data are expressed as frequencies and percentages, whereas continuous data are presented as either means and standard deviations or medians and interquartile ranges, depending on the distribution.

Subgroup analyses were performed to compare characteristics and treatment outcomes, using the chi-square test, the Mann–Whitney *U* test, and the Kruskal–Wallis test as appropriate. An alpha level lower than 0.05 was considered statistically significant. Both uni- and multivariate logistic regression were performed to investigate the relationship between survival and the studied variables.

## Results

The study analyzed 194 patients from 29 hospitals in 11 European countries, of whom 111 were men (57%), and 183 were Caucasian (94%). The most common ASA score was 3 (113 patients, 58%), and the median Charlson Comorbidity Index was 5 (interquartile range [IQR], 3–6). Additionally, 144 patients (80%) had an Eastern Cooperative Oncology Group (ECOG) performance score of 0–1, and the median body mass index (BMI) was 24.2 kg/m^2^ (Table 1).

**Table 1:** Data series

Baseline characteristics	
	(*n* = 194) *n* (%)
Gender	
Male Female	111 (57.2) 83 (42.8)
Age: years (IQR)	69 (62–75)
Ethnicity	
Asian Caucasian Latino Other	6 (3.1) 183 (94.3) 2 (1.0) 3 (1.5)
ASA	
I II III IV	14 (7.3) 113 (58.5) 62 (32.1) 4 (2.1)
ECOG	
0 1 2 3	74 (40.4) 70 (38.3) 29 (15.8) 10 (5.5)
Charlson comornidity index (IQR)	5 (3–6)
BMI: kg/m/^2^ (IQR)	24.2 (22–28.4)
Surgical history	
No Yes	126 (65.3) 67 (34.7)
Liver surgery Pancreatic surgery Cholecystectomy Other supramesocolic surgery Inframesocolic surgery	1 (1.5) 1 (1.5) 12 (17.9) 13 (19.4) 52 (77.7)
*Clinical, serologic, and radiologic data*	
Abdominal pain	
Yes No	104 (53.6) 90 (46.4)
Jaundice	
Yes No	84 (43.3) 110 (56.7)
Asymptomatic	
Yes No	16 (8.2) 178 (91.8)
Acute cholangitis	
Yes No	5 (2.6) 189 (97.4)
Weight loss	
Yes No	62 (32.0) 132 (68.0)
Bilirrubin: mg/dL (IQR)	0,8 (0,4–4,4)
Calcium: mg/dL (IQR)	8.7 (7.6–9.4)
CA 19.9: UI/l (IQR)	71 (19–276)
Hemoglobin: g/dL (IQR)	12.5 (11.6–13.5)
AST: U/L (IQR)	30 (20–79)
ALT: U/L (IQR)	36 (19–99)
Location	
Head	125 (64,8)
Other location	
Neck Body Tail Body-tail	7 (3,6) 25 (13) 28 (14,5) 8 (4,1)
Neoadjuvant chemotherapy	
Yes No	44 (22.7) 150 (77.3)
*Intraoperative data*	
Minimal invasive approach	
Yes Laparoscopic Robotic Hybrid No	17 (8.8) 12 (6.2) 3 (1.5) 2 (1.0) 177 (91.2)
Conversion	8 (4.4)
Type of resection	
Pancreatoduodenectomy Left pancreatectomy Total pancreatectomy	125 (64.4) 59 (30.4) 10 (5.2)
*Postoperative outcomes*	
Hospital stay: days (IQR)	13 (9–20)
Major complications (Clavien-Dindo ≥ IIIa)	49 (25.3)
Bile leak	
No Grade A Grade B Grade C	183 (94.8) 4 (2.1) 3 (1.6) 3 (1.6)
Hemorrhage	
No Grade A Grade B Grade C	169 (87.1) 9 (4.6) 7 (3.6) 9 (4.6)
Pancreatic fistula	
No Biochemical Grade B Grade C	146 (75.3) 23 (11.9) 24 (12.4) 1 (0.5)
Delayed gastric emptying	
No Grade A Grade B Grade C	167 (86.5) 15 (7.8) 7 (3.6) 4 (2.1)
Intraabdominal abscess	24 (12.4)
Medical complications	
Cardiac arrest Venous thromboembolism Stroke Pneumonia Urinary tract infection	7 (3,6) 2 (1.0) 1 (0.5) 4 (2.1) 4 (2.1)
Reintervention Interventional radiology Surgery Endoscopy	32 (16.5) 12 (37.5) 15 (46.9) 5 (15.6)
Readmission at 90 days	31 (16.0)
*Histologic data*	
Resection margin	
R0 R1 R1 vascular R2	125 (64.4) 53 (27.3) 11 (5.7) 5 (2.6)
Immunohistochemistry	
Ck5+ Ck6+ Ck7+ P63+ P40+ MUC1+	35 (18.0) 32 (16.5) 47 (24.2) 25 (12.9) 55 (28.4) 6 (3.1)
Size: mm (IQR)	35 (25–47)
Lymph nodes harvested	19 (12–28)
Vascular invasion	
No Yes	69 (35.6) 125 (64.4)
Lymphatic invasion	
No Yes	94 (48.5) 100 (51.5)
Perineural invasion	
No Yes	14 (15.1) 79 (84.9)

IQR, interquartile range; ASA, American society of anesthesiologists; ECOG, Eastern cooperative oncology group; BMI, body mass index; AST, aspartate aminotransferase; ALT, alanine aminotransferase

Of the 194 patients, 67 (35%) had a history of abdominal surgery, most frequently inframesocolic surgery (52 patients, 78%). The most common symptom reported was abdominal pain, experienced by 105 patients (54%). Laboratory findings were within the normal range for most individuals. The majority of the ASCPs (65%, 125 patients) had tumors located in the head of the pancreas, and only 44 (23%) received neoadjuvant chemotherapy.

Minimally invasive surgery was performed for 9% of cases (17 patients), with nearly 50% of the patients requiring conversion to an open procedure. We performed a total of 125 pancreaticoduodenectomies, 59 left pancreatectomies, and 10 total pancreatectomies.

Major complications (≥ 3a) occurred for 49 patients (25%). Specific complications related to pancreatic surgery included pancreatic fistulas in 48 patients (25%). Of these patients, 25 presented with clinically relevant pancreatic fistula −B+C− (13%), 26 presented with delayed gastric emptying (13%), 25 presented with postoperative bleeding (13%), and 10 presented with biliary fistula 10 (5%).

Reoperation was required for 15 patients (8%), and the 90-day readmission rate was 16%. The median hospital stay was 13 days (IQR, 9–20 days). The R0 resection rate was 64% (125 patients), and the median tumor size was 35 mm (IQR, 25–47 mm). The average number of harvested lymph nodes was 19 (IQR, 12–28). Vascular invasion was observed in 125 patients (64%), lymphatic invasion in 100 patients (51%), and perineural invasion in 79 patients (78%).

**Fig. 1:**
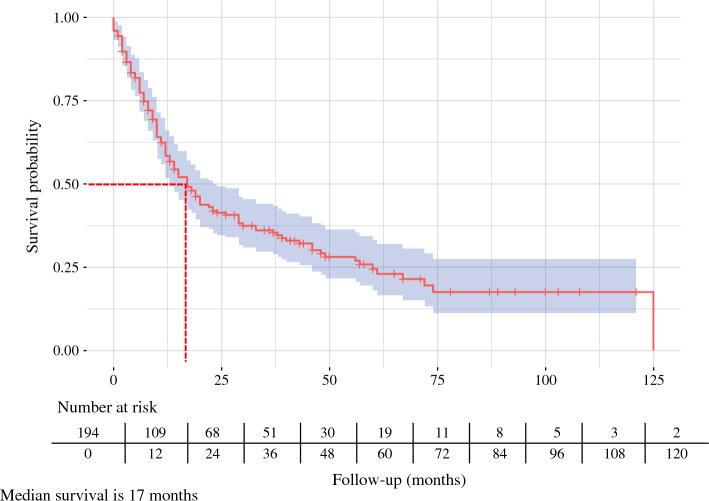
Overall survival among patients with resected adenosquamous carcinoma of the pancreas

**Fig. 2:**
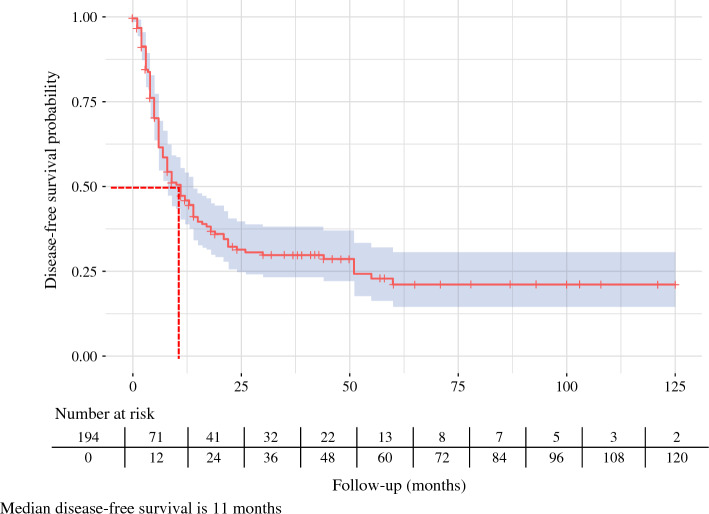
Disease-free survival among patients with resected adenosquamous carcinoma of the pancreas

The OS rates were 56% at 1 year, 26% at 3 years, and 10% at 5 years (Fig. 1). The DFS rates at the same intervals were 36%, 16%, and 7%, respectively (Fig. 2).

In the univariate analysis, factors associated with lower survival rates included any margin other than R0, a higher percentage of involved lymph nodes, lymphatic and vascular stromal invasion, T3–T4 stage of disease, no postoperative chemotherapy, and recurrence. In the multivariate analysis, shorter survival was observed to be associated with R2 resections, lymphatic stromal invasion, T4 stage disease, no adjuvant chemotherapy, and recurrence (Table 2).

**Table 2:** Uni- and multivariate regression about factors associated to survival

Variable	Univariate			Multivariate	
	1-Year OS (%)	HR (95% CI)	*P* Value	HR (95% CI)	*P* Value
*Baseline characteristics*					
Gender			0.106		
Male Female	54.9 57.8	Ref. 0.75 (0.52–1.06)			
Age		1.01 (0.99–1.03)	0.210		
ASA					
I	28.6	Ref.			
II	69.0	0.78 (0.34–1.82)	0.577		
III	40.3	1.29 (0.56–3.04)	0.547		
IV	50.0	1.29 (0.36–4.60)	0.693		
Previous surgeries					
No	57.9	Ref.			
Yes	53.7	1.04 (0.72–1.5)	0.822		
BMI		0.97 (0.93 –1.01)	0.060		
ECOG					
0	74 (40.4)	Ref.			
1	70 (38.3)	0.78 (0.34–1.82)	0.577		
2	29 (15.8)	1.29 (0.56–3.04)	0.547		
3	10 (5.5)	1.29 (0.36–4.60)	0.693		
Comorbidity Charlson Index		1.06 (0.99–1.13)	0.069		
*Clinical symptoms, location, and neoadjuvant treatment*					
Abdominal pain					
No Yes	61.1 51.9	Ref. 1.08 (0.76–1.52)	0.650		
Jaundice					
No	56.3	Ref.			
Yes	60.7	0.86 (0.61–1.22)	0.407		
Asymptomatic					
No	56.7	Ref.			
Yes	50.0	0.86 (0.46–1.61)	0.646		
Acute cholangitis					
No	56.6	Ref.			
Yes	40.0	2.06 (0.76–5.59)	0.550		
Weight loss					
No	50.7	Ref.			
Yes	67.7	0.98 (0.68–1.41)	0.899		
Location					
Head	56.8	Ref.			
Neck	42.8	1.02 (0.37–2.78)	0.975		
Body	76.0	0.62 (0.34–1.13)	0.120		
Tail	42.8	1.37 (0.85–2.20)	0.202		
Body-tail	37.5	2.15 (0.99–4.67)	0.052		
Neoadjuvant chemotherapy					
No	55.3	Ref.			
Yes	59.0	0.96 (0.63–1.45)	0.839		
*Intraoperative*					
Minimally invasive approach					
No	57.1	Ref.			
Yes	47.1	0.96 (0.52–1.79)	0.904		
Bleeding					
No	55.9	Ref.			
Yes	100.0	0.96 (0.13–6.89)	0.969		
Type of resection					
Pancreatoduodenectomy	58.4	Ref.			
Left pancreatectomy	54.2	1.13 (0.77–1.69)	0.542		
Total pancreatectomy	40.0	1.71 (0.82–3.54)	0.152		
*Postoperative events*					
Hospital stay (days)		0.99 (0.99–1.00)	0.550		
Complications Clavien ≥ IIIa					
No	55.9	Ref.			
Yes	57.1	1.21 (0.81–1.79)	0.351		
Reintervention					
No	56.8	Ref.			
Yes	53.1	1.14 (0.71–1.82)	0.595		
Readmission at 90 days					
No	57.7	Ref.			
Yes	48.4	1.33 (0.86–2.06)	0.198		
*Histology*					
Resection margin					
R0	66.4	Ref.		Ref.	
R1 and R1 vascular	40.6	2.03 (1.42–2.90)	**< 0.001**	1.59 (0.98–2.59)	0.057
R2	0.0	10.63 (4.13–27.37)	**< 0.001**	9.89 (2.53–38.7)	**0.001**
Diameter (maximum)		1.02 (1.01–1.03	**< 0.001**	1 (0.99–1.02)	0.206
No. of lymph nodes involved		1.03 (1.01–1.06)	**0.040**	0.98 (0.95–1.03)	0.562
Stromal vascular invasion					
No	63.7	Ref.		Ref.	
Yes	50.0	1.72 (1.17–2.51)	**0.005**	0.93 (0.58–1.49)	0.775
Lymphatic invasion					
No	63.7	Ref.		Ref.	
Yes	50.0	1.83 (1.29–2,61)	**0.001**	1.68 (1.1–2.58)	**0.017**
Perineural invasion					
No	64.3	Ref.			
Yes	53.9	1.38 (0.89–2.12)	0.148		
T					
T1	77.3	Ref.		Ref.	
T2	53.1	1.29 (0.66–2.53)	0.451	1.14 (0.55–2.35)	0.717
T3	54.5	2.16 (1.17–3.97)	**0.014**	1.66 (0.79–3.47)	0.180
T4	44.4	3.56 (1.45–8.75)	**0.006**	3.03 (1.01–9.14)	**0.049**
Positive vascular margin					
Yes	43.2	Ref.		Ref.	
No	60.0	0.54 (0.36–0.81)	**0.003**	1.04 (0.61–1.77)	0.898
Positive retroperitoneal margin					
Yes	47.9	Ref.		Ref.	
No	60.6	0.51 (0.35–0.75)	**0.001**	1.15 (0.7–1.88)	0.579
Unknown	0.0	1.74 (0.41–7.27)	0.451	1.15 (0.98–21.36)	0.053
*Postoperative treatment*					
Adjuvant chemotherapy					
Yes	39.4	Ref.		Ref.	
No	64.8	1.89 (1.33–2.70)	**<0.001**	2.05 (1.37–3.05)	**<0.001**
*Recurrence*					
Recurrence					
No	57.7	Ref.		Ref.	
Yes	57.1	2.79 (1.79–3.35)	**<0.001**	2.7 (1.64–4.45***)***	**<0.001**

OS, overall survival; HR, hazard ratio; CI, confidence interval; ASA, American Society of Anesthesiologists; BMI, body mass index; ECOG, Eastern Cooperative Oncology Group

The results highlighted in bold are those that are statistically significant

## Discussion

Compared with PDAC, ASCP is known to be an aggressive tumor associated with poorer survival rates due to characteristics such as larger size, lymph node involvement, greater vascular invasion, and lower differentiation.^2,6,7,11^ Interestingly, however, the resectability rate for ASCP is higher than for PDAC.^2,3,5–7^ In our study, the overall 5-year survival rate was 9.8%, and the DFS rate was 0.1%, both of which are somewhat lower than those reported in PDAC studies.^2,3,5–8^ Published 5-year survival rates for patients with ASCP range from 7 to 18%, and the rates in the current study fall within this range.

In our series of 194 resected ASCPs, it is noteworthy that the most common tumor location was the pancreatic head. This finding contrasts with results from other published studies, in which the body and tail were the most frequent locations. We have no clear explanation for this discrepancy.^1,3^ The median age was 69 years, with a slight predominance in males, as observed in most published series.^1,3^ We also observed postoperative results, including overall and specific complications, resection status, and number of lymph nodes harvested, which were like those reported in series focused on PDAC. Additionally, the low percentage of minimally invasive approaches in our series, likely related to the larger size of ASCP versus PDAC, warrants further consideration.^1,3,7,11^

The prognostic factors for ASCP include age, gender, race, tumor size, use of radiotherapy or/and chemotherapy, preoperative levels of CA19-9, surgery, lymph node ratio, tumor differentiation, M1, and the anatomic site of the tumor being worse in the head of the pancreas.^2,7–9^ Some researchers propose that the presence of the squamous element is a negative prognostic factor.^1^

The benefits of neoadjuvant and adjuvant chemotherapy for ASCP patients remain unclear because most available data predate the introduction of modern multi-agent chemotherapy.^2,5^ Nevertheless, some studies indicate that platinum-based chemotherapy in the adjuvant setting may increase overall median survival.^10,17^ In our series, some previous factors such as tumor size (T4) were confirmed. However, we also recognized additional factors such as R2 resections, lymphatic invasion, and postoperative recurrence.

Between 60 and 75% of patients with ASCP receive postoperative chemotherapy. Several factors can prevent patients from receiving chemotherapy, including severe postoperative complications, age (specifically age older than 75 years), and existing comorbidities.^5,10^ This is significant because combining surgery with chemotherapy has been linked to improved survival rates, but not all patients complete this treatment.^5,10^ In our study, individuals who did not receive chemotherapy also exhibited poorer survival rates. Currently, there is no evidence to support a general recommendation for neoadjuvant chemotherapy.^5^ It has been suggested that stromal invasion by tumor and cellular budding are associated with a poorer prognosis in PDAC. Studies have indicated that ASCP exhibits higher rates of lymphatic, vascular, and perineural invasion, which may contribute to a worse prognosis compared with typical pancreatic cancer.^29,30^ In our study, the multivariate analysis identified lymphatic stromal invasion as a negative prognostic factor.

The limitations of this study were those characteristic of a multicenter analysis that includes various therapeutic approaches across different participating centers, and the histology was not independently centrally re-reviewed. However, the strengths of the study were in the large patient sample of approximately 200 individuals and the inclusion of very recent cases, up to 2024, which adhere to current quality standards for pancreatic surgery.

In conclusion, patients who undergo resection for ASCP experience very low survival rates (~10%). It is advisable to avoid resection of T4 tumors, patients with significant comorbidities, and R2 resections because these factors are related to poorer survival outcomes. Additionally, a failure to provide adjuvant chemotherapy, often due to severe postoperative complications, further deteriorates the prognosis. Neoadjuvant chemotherapy could perhaps help to improve these dismal results.
